# Supplementary material for: Are carbon emissions trading and green financial instruments synergistic? -Comprehensive quantitative research based on content analysis
Source: PLoS One. 2024 Mar 7;19(3):e0298601. doi: 10.1371/journal.pone.0298601 (PMC10919601; doi:10.1371/journal.pone.0298601)
Supplement: S1 Data — (ZIP) [file pone.0298601.s001.zip › Data/地级市Z_F工作报告.zip!/─■╧─/╣╠╘¡╩╨/└┤╫╘╣1⁄2╓┌║┼í╛┬φ┐╦╩2╛▌═°í┐_2019.docx]

各位代表：

我代表市人民政府向大会报告工作，请予审议，并请市政协委员和列席人员提出意见。

2018年工作回顾

2018年，我们迎来了改革开放40周年，40年的伟大成就和宝贵经验，极大地鼓舞着我们坚定不移将改革开放进行到底；我们迎来了自治区成立60周年，60年的伟大变革和成功实践，更加坚定了全市各族干部群众感党恩听党话跟党走的如磐信念。

一年来，市人民政府深入学习贯彻习近平新时代中国特色社会主义思想和党的十九大精神，把习近平总书记视察宁夏时的重要讲话作为全面落实自治区第十二次党代会和市委四届二次、三次全会精神的根本遵循，坚持新发展理念，统筹做好稳增长、促改革、调结构、惠民生、防风险各项工作，全市经济运行稳中有进、各项事业稳步向好。完成地区生产总值303.2亿元，可比增长6.6%；完成地方一般公共预算收入17.3亿元，同比增长3.5%；全社会固定资产投资的降幅从二季度起逐季收窄；社会消费品零售总额73.7亿元，同比增长3.8%；城乡居民人均可支配收入分别达到26709元、9557元，分别增长8.4%和11.4%。一年来，全市人民在市委的坚强领导下，攻坚克难，砥砺前行，创造了新成绩、展现了新亮点：固原市被命名为“全国民族团结进步创建示范市”，“四个一”林草产业试验示范工程得到中央领导充分肯定，创办的新时代农民“讲习所”被中宣部确定为基层宣传工作典型案例，“金融扶贫”机制被国务院扶贫办向全国推广，“331”监管平台入选“中国网络理政十大创新案例”。

一年来，我们主要抓了以下七个方面工作：

一是把政治建设摆在首位。自觉在思想上政治上行动上同以习近平同志为核心的党中央保持高度一致。提高政治站位，忠诚于党。把习近平新时代中国特色社会主义思想和党的十九大精神作为政治必修课，教育政府系统各级党组织和党员干部树牢“四个意识”，坚定“四个自信”，做到“两个维护”。坚持党对一切工作的领导，坚决落实党中央、国务院和自治区党委、政府及市委决策部署，制定落实中央“三大攻坚战”行动方案，推进自治区“三大战略”和固原“六场硬仗”、“四个示范市”建设落地落实。坚持党组中心组学习等制度，全年召开市政府党组会议20次、班子成员下基层调研累计460次，做到了学理论、解民忧、谋发展互促共进。对标整改问题，行成于思。对中央第八巡视组、中央第二环保督察组“回头看”及国务院第五次大督查反馈问题照单全收，按照市委“1+8”整改方案，制定了贯彻新发展理念和中央重大决策部署的问题、任务、责任“三个清单”，市政府班子成员带头整改，各部门、各县（区）认真履职，按进度完成了整改内容，取得了阶段性成效。改进工作作风，取信于民。扎实开展脱贫攻坚作风建设年、干部素质提升年、干部作风转变年活动，推动解决干部工作能力不足和责任担当不够等问题。全年办结区、市人大代表议案建议38件、政协提案148件，提请市人大常委会审议通过地方性法规2件。全面推进政务公开，彭阳县被确定为全国基层政务公开标准化规范化试点单位。认真履行市政府党组全面从严治党主体责任，坚决开展违反中央八项规定精神突出问题专项治理，查处违规问题89起，处理党员干部155人，党纪政务处分86人；查处扶贫领域腐败和作风问题240起，处理365人。

二是竭力打好脱贫富民决胜战。销号贫困村58个、减贫5.2万人，贫困发生率下降到3.7%。隆德、泾源、彭阳具备贫困县脱贫退出条件。产业扶贫提质增效。扶持发展养殖专业合作社33个、龙头企业52家，建成电商示范村10个。培训劳动力4.38万人，打造区级众创空间2个、市级创业孵化基地3个，培育创业实体1596户，带动就业9892人，累计转移就业31.8万人，创收66.4亿元。设施短板对标补齐。整合涉农资金37.6亿元，新改建农村公路400公里、村组巷道689公里，新增自来水用户1.25万户，改造危房13990户。搬迁安置移民50939人，基本完成“十三五”移民任务。金融扶贫普惠利民。新增金融机构3家，全市金融机构存贷比92.8%，较年初上升9.7个百分点。建立各类担保基金14.1亿元，撬动银行贷款61.3亿元，扶持贫困户、涉农企业、“两个带头人”发展壮大。创建金融扶贫示范村100个，建档立卡户户均贷款5万元，覆盖率86.7%。闽宁协作务实深入。落实闽宁帮扶资金1.88亿元，实施扶贫项目65个，建设闽宁示范村36个；援建扶贫车间63个，帮助11396名贫困人口就业。加强闽宁扶贫协作交流，结成部门、乡镇、村“一对一”帮扶120对。61家福建企业来固投资20.67亿元，带动3790名贫困人口脱贫。脱贫动力外激内生。支持“两个带头人”工程不断提升，新培育致富带头人626名；开展农民培训教育年活动，培训农民25.8万人次；选树表彰“脱贫光荣户”，激励群众不等不靠，苦干实干。涌现出全国脱贫攻坚先进个人樊学双、齐永新和全区脱贫攻坚优秀共产党员马新娟、撒凤虎等先进典型。

三是扎实打好创新驱动攻坚战。全市R&D投入1.46亿元，支持科技创新，促进产业发展。农业接二连三。粮食总产84.2万吨，创历史新高。制定优势特色产业提质增效实施方案，成立国家肉牛改良中心固原试验示范站，肉牛饲养量110万头，其中安格斯肉牛4.8万头；发展绿色冷凉蔬菜51万亩；落实习近平总书记做实做强“中国马铃薯之乡”的指示精神，突出品种调整和主食化开发，种植马铃薯125万亩，加工销售马铃薯饼干、粉条等主食化产品1586吨。西吉县被评为全国十大马铃薯主食化示范基地，泾源黄牛肉、别庄牌菜心获中国国际农交会金奖，六盘山高原亚麻籽油摘得中国油博会金奖。建立“四个一”林草产业试验示范园（点）39个2.1万亩，引进149种树（草）、试种303个品种，初步筛选出大果榛子、海棠、黑果花楸、波斯菊等适宜固原栽植的品种；打造休闲农业经营主体35家。工业延链补链。金昱元40万吨高性能聚氯乙烯树脂、丰源20万锭纺纱一期等9个项目建成投产，王洼煤矿600万吨改扩建项目建成试运行。出台工业园区优化提升和改革创新行动方案、促进民营经济加快发展若干意见，为51家企业落实贷款1.43亿元，争取自治区奖补资金1亿元，帮助中小企业挖潜升级。新增规上工业企业15家，培育国家高新技术企业3家、国家科技型中小企业6家。服务业稳中有进。围绕创建全国旅游标准化试点城市，完成六盘山长征纪念馆等景区基础改造和旅游驿站、慢行系统等旅游环线配套设施建设，西吉县龙王坝村创建为3A级景区，彭阳县长城村乔渠组入选中国传统村落名录。与重庆市签订旅游合作框架协议，加入陕甘川宁毗邻地区旅游合作联盟，全年接待游客550万人次、旅游社会收入40亿元，分别增长12%、30%。新增服务业企业132家、商贸流通企业15家。电子商务和物流快递融合发展，农特产品线上销售2.2亿元。

四是集中打好生态环境保卫战。清水河水污染问题被环保部约谈后，我们坚持问题导向，铁腕治污，坚决守护绿水青山。开展全域绿化行动。完成六盘山重点生态功能区造林65万亩，绿化城乡环境7.5万亩、道路505公里；治理水土流失面积350平方公里；完成自然保护区生态修复5.14万平方米。森林覆盖率达到26.8%。落实污染综治措施。压紧靠实河长制，投资6.2亿元，综合治理五河流域水环境，出境断面水质均为Ⅳ类及以上，河长制考核居全区五市第一。农业面源污染治理取得新成效，畜禽养殖废弃物综合利用率93.6%，农残膜回收率96%。大气污染防治巩固提升，完成西吉、隆德县城供热锅炉脱硫脱硝改造、市区1322家餐饮单位煤改气、煤改电任务，PM2.5同比下降3.1%，空气优良天数达96.5%，同比提升0.8%，环境质量考核居全区五市第一。

五是协力打好城乡统筹大会战。推进城乡基础设施互联互通、提档升级。城市基础功能提升。改造市区棚户区6100余户。实施海绵城市项目44个，海绵化改造老旧小区14个、城市公园3个，完成文化路、中山街等雨污分流改造；新增城市绿地24.7万平方米。完成西吉体育馆、隆德长乐街雨污分流改造、泾源高级中学扩容改造、彭阳雷河滩全民健身中心等县城基础设施提升项目，城市综合承载和服务保障功能不断增强，全市常住人口城镇化率49.8%。启动实施“九城同创”工作。乡村振兴内外兼修。制定实施乡村振兴战略意见，先行启动农村人居环境整治三年行动，以“四清四改”为抓手，改造农村厕所1.27万座，建成乡村污水处理站27个。实施5个美丽小镇和42个美丽村庄建设项目。泾源县冶家村评选为“2018年中国美丽休闲乡村”。西吉县红耀乡井湾村、泾源县香水镇上桥村、彭阳县红河镇被认定为全国一村一品示范村镇。G327线沟圈至彭阳建成通车，S25线泾河源至华亭、S60线西吉至会宁建设加快推进。隆德县城乡供水工程竣工通水，新建、改造西吉王民、彭阳石头崾岘等水库12座，发展高效节水灌溉面积7.2万亩。完成新一轮农网改造升级工程，解决了2万余户10万多群众用电“卡脖子”问题。建成4G基站375座，1087个自然村通光纤宽带。

六是用情打好民生改善持久战。财政支出重点用于民生事业，8项16件民生实事基本办结。社会事业全面进步。新（改）建幼儿园32所，新增园位3200个，固原八中建成招生，新增学位1500个，固原一中初中部改扩建项目投运；西吉县义务教育均衡发展通过国家评估认定。宁夏师范学院新增为硕士学位授予单位。实施医疗卫生人才“双优”工程，组建大专家医学教育院士工作站、韦以宗名医工作室；与北京宣武、西京等区内外14家三甲医院建立远程诊疗，县域内就诊率87.6%。彭阳县荣获全国“互联网+健康扶贫”实践奖。国家公共文化服务体系示范区创建工作全面启动，固原特色文化产业“一园一馆一中心一遗址”等3个项目入选国家文化和旅游部《2018文化产业项目手册》，彭阳姚河塬西周遗址入选“全国十大考古新发现”，固原砖雕、隆德杨氏泥塑入选国家传统工艺振兴目录，马金莲短篇小说《1987年的浆水和酸菜》获第七届鲁迅文学奖。社会保障兜牢底线。新增城镇就业8982人，城镇登记失业率3.69%。社会保险扩面提标，建档立卡贫困人口养老、医疗保险参保率均达到100%，城乡居民基础养老金从每人每月135元提高到158元。隆德县入选全国首批社会救助综合改革试点县。建成固原市养老综合服务中心，新建农村老饭桌183个、农村（社区）养老机构35个，更好地满足了老年人的医养服务需求。社会大局和谐稳定。全市政府显性债务余额112.6亿元，控制在自治区核定限额内；开展P2P网络借贷风险专项整治，立案查处非法吸存案件11起，涉案金额2.46亿元。创新丰富“拥护核心感党恩，同心携手奔小康”主题教育活动，促进各民族交往交流交融；出台民族宗教领域突出问题整改工作指导意见，依法加强宗教事务管理，“三化”治理稳妥有序推进。深入开展平安固原建设，“七五”普法中期评估通过自治区考核验收，泾源县荣获全国法治县创建活动先进单位；安全生产形势总体稳定，食品药品监管不断加强；扫黑除恶专项斗争全面开展，立案侦查涉黑涉恶案件21起，打掉涉恶团伙9个。公众安全感连续八年位居全区五市第一。

七是创新打好改革开放突围战。以全面深化改革破解发展瓶颈，以全方位开放拓展发展空间。改革红利不断释放。深化“放管服”改革，办事材料减少30%以上，90.6%的事项网上可办，81.7%的事项可不见面办理。农村土地承包经营权确权100%，抵押土地2.1万亩，贷款1536笔1776.4万元。隆德县被确定为全国农村集体产权制度改革试点县。开放领域不断拓展。积极融入“一带一路”建设，成功举办陕甘川宁经联会，积极参加西交会、宁商大会等。修订招商引资优惠政策，全年招商引资到位资金131亿元。

统计调查、防震减灾、档案、残联、地方志、工青妇、国防动员和民兵预备役等工作得到新加强、取得新成效。

各位代表，回顾过去的一年，我们体会到，习近平新时代中国特色社会主义思想是固原决战脱贫攻坚、决胜全面小康的行动指南，忠诚践行“四个意识”和“两个维护”的思想、政治、行动自觉，自治区党委、政府和市委的坚强领导、把舵定向是做好政府工作的政治保证，市人大及其代表的法律监督与支持、市政协及其委员的民主监督与帮助是政府正确履职的重要保障，广大干部群众齐心协力、苦干实干是政府各项工作取得实效的力量源泉。在此，我代表市人民政府，向各位代表、各位委员，向各民主党派、工商联、无党派人士和各人民团体，向中央、自治区驻固单位、驻固部队、武警官兵和公安干警，向关心支持固原发展的社会各界，表示衷心的感谢！

总结过去，展望未来，发展不充分不平衡的一些问题尚未有效解决，政府自身建设还存在许多不足。一是对标中央和区、市党委决策部署在落实落细上有差距。运用习近平新时代中国特色社会主义思想指导实践、推动工作的能力不强。贯彻新发展理念与实际工作结合不紧密，创新精神、开放思维、市场意识不强。受项目接续储备不足、推进工作不力、市场培育滞后等因素影响，地区生产总值、固定资产投资、社会消费品零售总额没有完成预期；产业项目支撑能力弱，新产业新业态培育不足，推动高质量发展任重道远。二是对标人民对美好生活的向往在解决问题上有差距。群众稳定持续增收基础不牢固，农民经营性收入不稳定，技能培训不精准，劳务组织化程度不高。扶贫帮扶责任落实不深不细。因前期论证不充分、项目计划调整，3件民生实事未能按期完成，其中社区卫生服务中心停止实施、改扩建幼儿园和海绵化改造老旧小区项目推进缓慢。基础设施、公共服务领域还有不少短板，社会治理、污染防治等还存在薄弱环节，同步建成全面小康社会任务十分艰巨。三是对标全面从严治党要求在改进作风上有差距。表态调门高、行动落实差，重“痕”不重“绩”、留“迹”不留“心”，不担当、不作为等问题不同程度存在。有的干部工作上粗枝大叶，得过且过，解决群众诉求没耐心、不用心，有始无终；主动服务企业和群众意识不强，营商环境亟需优化。违反中央八项规定精神和扶贫领域腐败问题时有发生。我们一定要正视差距，直面问题，主动作为，不辱历史使命，不负人民重托。

2019年主要工作任务

按照市委四届四次全会部署，政府工作的总体要求是：以习近平新时代中国特色社会主义思想为指引，以习近平总书记视察宁夏时的重要讲话精神为根本遵循，全面贯彻党的十九大、十九届二中、三中全会和中央经济工作会议精神，统筹推进“五位一体”总体布局，协调推进“四个全面”战略布局，全面落实自治区第十二次党代会、十二届六次全委会精神，坚持稳中求进总基调，坚持新发展理念，坚持推进高质量发展，坚持以供给侧结构性改革为主线，坚持深化市场化改革、扩大高水平开放，紧紧围绕“经济繁荣、民族团结、环境优美、人民富裕”目标，以脱贫攻坚统领经济社会发展全局，坚决打好“三大攻坚战”，深入实施“三大战略”，集中攻坚“六场硬仗”，加快建设“四个示范市”，振奋精神、实干兴宁，为打赢脱贫攻坚战、全面建成小康社会打下决定性基础，以优异成绩庆祝中华人民共和国成立70周年。

主要目标是：全市地区生产总值增长7%，地方一般公共预算收入同口径增长7.5%，全社会固定资产投资增长5%以上，社会消费品零售总额增长6%，城乡居民人均可支配收入分别增长8%、9%；完成自治区下达的各项约束性指标。

各位代表，完成这些目标任务，关键在于深入贯彻中央经济工作会议精神，坚持好“五个必须”，落实好“八字方针”，坚定信心，勇往直前，不断推动高质量发展。重点抓好以下工作：

一、突出抓农民稳定增收，持续推进脱贫富民决胜战，让老百姓的满意度更高。坚决打好精准脱贫攻坚战，深入实施脱贫富民战略，实现21个贫困村脱贫出列、减贫4万人，抓好隆德、泾源、彭阳三县脱贫摘帽迎检考核工作，争取原州区具备脱贫退出条件，西吉县脱贫工作取得决定性进展。集中攻坚深度贫困村。聚焦“两不愁三保障”，逐户查漏补缺，逐项巩固提升；应用扶贫云“一点通”，把好脱贫管理、评估、销号三道关口，坚决防止算账式脱贫；整合涉农资金40亿元，改造危房6555户，新修“四好农村路”500公里，实施7项农村人饮巩固提升工程，建成扶贫车间50个，推动基础设施和公共服务等重点任务销号清零，促进贫困村和非贫困村协调发展。壮大脱贫富民产业。提高“有土”扶贫产业化水平，规范发展新型农业经营主体150个、休闲农业经营主体15个、乡村旅游示范村13个，让贫困户、非贫困户与产业紧密联结。提高“离土”扶贫组织化程度，精准培训劳动力3万名，转移就业30万人以上，创收65亿元以上。发展金融扶贫示范村200个，实现贫困户和脱贫户产业贷款应贷尽贷，帮助边缘群体稳定增收。压紧靠实帮扶责任。深入推进脱贫攻坚作风建设年活动，有效解决政策、工作、责任落实不严不实问题。贯彻全国东西部扶贫协作座谈会和闽宁扶贫协作会议精神，推动两地联系更密、合作更实。深化“两个带头人”工程，新培育农村致富带头人300名；深入开展农民教育培训年活动，丰富新时代文明实践中心内容载体，通过典型示范、能人带动、教育激励、政策引导，提振贫困群众奋发自强、实干脱贫的精气神。

二、突出抓产业融合提质，持续推进创新驱动攻坚战，让老百姓的日子更好。认真落实稳就业、稳金融、稳投资、稳预期工作要求，坚持以供给侧结构性改革为主线，深入实施创新驱动战略，全面落实“巩固、增强、提升、畅通”方针，加快推动特色农业接二连三、生态工业提效升级、现代服务业提速扩量。巩固来之不易的成果。坚决用市场化、法治化手段淘汰落后、低端、过剩产能，促进优质资源向优势企业集聚。全面落实国家和自治区新一轮减税降费政策和降成本措施。实施工业园区低成本化改造，研究出台降低物流成本意见，最大限度降低企业经营性成本、挤压制度性成本，让利于企、放水养鱼。增强民营经济活力。全面落实自治区民营经济“20条”，创造公平竞争的市场环境，保障民营企业在市场准入、合法经营、公共资源交易等方面的正当权益；密切政企沟通联系，帮助企业纾困解难，构建亲清新型政商关系。加快工业园区优化提升和改革创新，理顺体制机制、完善服务功能，培育规上工业企业10家以上。继续加大政府科技投入，落实科技扶持资金5000万元，引导金融支持科技型企业发展，R&D投入强度达到0.5%以上，科技型中小企业突破100家。提升产业链水平。农业主抓深加工和外销，重点力促10万头肉牛精深加工和生态养殖园区等重大项目落地，加快马铃薯循环产业园项目、植物蛋白饲料、港生中药材种加销“一体化”项目建设，高质量培育“5个10亿农业产业集群”。工业突出无中生有、固原所需，重点加快丰源20万锭纺纱二期、天楹垃圾发电、双文绒业羊绒制品等项目建设，争取彭阳鑫卓能源煤矸石加工等项目开工。服务业围绕人民生活新需求、消费新热点，重点抓好城市客厅、旅游综合体、医疗康养项目和义乌商品城建设，培育信息服务、健身娱乐、教育培训等新的消费增长点。推进国家全域旅游示范区建设，争取六盘山红军长征旅游区成功创建5A级、泾源兴盛滑雪场等冬季旅游项目建成运营。全年接待游客650万人次，实现旅游社会收入45亿元。畅通经济血液循环。围绕既有工业企业加快延链补链强链，实现从原材料、中间体到成品、副产品全部“吃干榨净”；打造集循环农业、创意农业、农事体验为一体的旅游小镇2个、乡村旅游田园综合体3个和市区旅游休闲综合体。推进“引金入固”计划，出台信贷担保、权益保护等金融惠企政策，支持鼓励金融机构加大对民营企业的信贷投放，调动“政银企”三方力量共同缓解企业融资难题。做好“国字头”六盘山农产品品牌打造和营销。

三、突出抓综合治理修复，持续推进生态环境保卫战，让老百姓的生活环境更美。坚决打好污染防治攻坚战，深入实施生态立区战略，为建设美丽新宁夏持续奋斗。推广“四个一”示范工程。围绕“一屏一带一线三区五城”的总体布局，出台扶持政策，引导企业主体、市场主导发展林草产业，打造示范点36个，试验示范“四个一”林草产业6万亩，推广“一棵草”60万亩，加快试验示范成果转化，走出生态美、产业兴、百姓富的多赢路子。为美丽固原增添新绿。统筹山水林田湖草系统治理，实施五县区造林绿化和火石寨自然保护区生态修复、三关口矿山地质环境治理等项目，高质量完成六盘山重点生态功能区造林绿化65万亩，打造3000亩以上集中连片示范点10个，绿化道路200公里，治理水土流失面积260平方公里。实施市区“四路两园一河一广场”和各县绿化提升工程，巩固国家园林城市创建成果。提升生态环境质量。持续开展蓝天、碧水、净土行动，一以贯之推动中央环保督察反馈问题整改到位。严格落实河长制，深入推进五河流域综合治理，实施清水河市区段水环境综合整治、葫芦河湿地恢复、渝河排污管网等水污染防治项目，实现水质稳定达标。坚持“四尘同治”，推动大气污染预控预警和联防联控，完成各县清洁煤配送中心、彭阳县集中供热热源环保设施改造等项目，空气质量优良天数稳定在90%以上。综合防控农业面源污染，确保土壤环境质量总体稳定。

四、突出抓功能完善提升，持续推进城乡统筹大会战，让老百姓的获得感更强。统筹做好城乡规划、精品建设、精细管理，让城乡的“面子”更美丽、“里子”更健康。增强城市承载能力。开展城市建设管理提升年活动，年内高质量修编城市总规、控规及城市重点建筑设计。改造市区棚户区1000户、老旧小区10个，完善12条城市道路及配套设施，改扩建东环路、机场至上海路等安全干线公路5条118公里，亮化美化城市重点街区、山体、水系，加快生态园林和文化旅游城市建设。完成海绵城市项目52个，努力建成黄土高原地区海绵城市建设示范市。开工建设固原市黄河水资源配置工程，形成黄河水、泾河水互补联调的安全供水网络。新建市区集中供热热源，让每一位市民温暖过冬。进一步理顺城市管理体制机制，改进社区物业服务管理，有效治理城市管理乱象，统筹推进“九城同创”，力争创成全国双拥模范城市、国家卫生城市。实施西吉、隆德、泾源、彭阳县棚户区和老旧小区改造、城市停车场、街巷道路建设等项目，进一步完善县城功能。实施乡村振兴战略。按照乡村振兴战略规划，重点推进产业振兴，集中打造草畜产业示范点55个、马铃薯产业示范基地10个、冷凉蔬菜产业示范基地16个、小杂粮示范基地30个、示范蜂场58个，培育壮大区域性特色产业，增加优质绿色农产品供给。重点推进文化振兴，充分发挥“一约四会”等制度作用，淳化民俗民风，限制高额彩礼，破除厚葬薄养等陈规陋习和不良行为，焕发文明新风。重点推进生态振兴，精心打造将台、孟塬等美丽小城镇7个和韩寨、胡大堡等美丽村庄31个；聚焦“四清四改”，改造农村厕所1.65万座，推动农村面貌焕然一新。

五、突出抓服务群众效率，持续推进民生改善持久战，让老百姓的烦心事更少。民生无小事，枝叶总关情。坚持以人民为中心的发展思想，尽心尽力办实事、解难事。发展优质均衡的教育事业。新建改建城乡幼儿园24所、中小学校25所。加快“互联网+教育”建设，推进“十百千”名师培养计划，助推教师素质、教育质量“双提升”。理顺市区教育管理体制，规范支持民办教育发展。支持宁夏师范学院建设“西部一流”学科和高水平本科教育，不断提高人才培养质量。提供优质便捷的医疗服务。推进健康固原建设，加快城市医联体、县域医共体建设，借助“互联网+医疗健康”，开展基层检查、上级诊断远程医疗服务，让群众看病更方便。建成投运市中医院改扩建等项目。建立名医工作室6个、重点优势特色科室12个，加速医师素质和医疗质量“双提升”。构建公平可靠的社会保障。培育创业实体1500个，新增城镇就业8000人，城镇登记失业率控制在4.5%以内。提高城乡低保、养老待遇和社会救助水平，解决好城乡群众生活困难问题。深化军民融合发展，加强退役军人事务管理和帮扶工作。供应丰富多彩的文化产品。实施公民道德建设工程，深化群众性精神文明创建活动，引导广大群众自觉践行社会主义核心价值观。推进国家公共文化服务体系示范区创建工作，提升22个乡村文化大院。办好中华人民共和国成立70周年系列庆祝活动和第五届宁夏六盘山登山节等文体活动，不断满足广大群众的精神文化需求。营造和谐稳定的社会环境。落实领导包案化解重点信访问题制度，解决群众反映强烈的突出问题。扎实开展“七五”普法，持续推进扫黑除恶专项斗争及社会乱象整治专项行动。严格地方党政领导干部安全生产责任制规定及实施细则，加强食品药品安全监管，坚决遏制重特大安全事故发生。持续深化马克思主义民族观宗教观教育，深入开展“拥护核心感党恩、同心携手奔小康”主题教育活动，筑牢中华民族共同体意识，巩固提升全国民族团结进步创建示范市成果，争取西吉、隆德创建为全国民族团结进步创建示范县。牢牢把握宗教中国化方向，依法管理宗教事务，引导宗教与社会主义社会相适应。

六、突出抓营商环境优化，持续推进改革开放突围战，让老百姓办事更快。深入学习贯彻习近平总书记在庆祝改革开放40周年大会上的重要讲话精神，坚定改革信心，破解制约瓶颈，释放发展活力。开展“优化营商环境”专项行动。全面落实习近平总书记在民营企业座谈会上提出的6条政策措施和国办优化营商环境“26条”，建立公平公正、开放透明的市场规则和法治化营商环境；刀刃向内破除审批慢、环节多、服务差等顽疾，让企业家专心创业、放心投资、安心经营，涵养出营商环境的“清新空气”。深入推进“放管服”改革，全面推开“一网、一门、一次”改革，市、县（区）政务服务事项网上可办率稳定在80%。推广投资项目审批“多评合一”“区域评”等改革经验，企业投资项目审批时间压缩到45个工作日之内。深化关键领域改革。完成机构改革任务。全面完成农村宅基地和房屋统一确权登记颁证工作，建成农村产权抵押贷款示范村10个、农村土地股份制改革示范点10个、农村集体资产股权权能改革示范点19个，推进农村土地适度规模经营，让农民享受到更多土地增值收益。建成“智慧固原”一期项目，推动大数据、物联网、云计算等现代技术在城市管理、社会治理、政务服务等领域融合应用。改革完善国有资产授权经营和监督管理体制，加快从管企业向管资本转变，提高“4+1”国有集团公司实体化、市场化运营水平。深化统计管理体制改革，强化依法治统，确保数据质量。持续扩大开放合作。深度融入“一带一路”和宁夏内陆开放型经济试验区，深化与东部沿海、省际毗邻等地区交流合作；办好第28届中国西部商品交易会，促进特色产品走向国内外大市场。开工建设宝中线固原至中宁南段扩能改造及固原火车站改扩建项目，早日实现固原人民期盼已久的“动车梦”；力争开工G85线银昆高速固原段项目；实施六盘山机场扩建停机坪、南侧GPS起降控制工程，畅通城乡内外大通道。开展“高质高效招商引资年”活动，强化园区和县区招商引资主体责任，紧盯重点领域、优质企业，全力推进精准招商，提高项目落地率、资金到位率、投产达标率，全年招商引资到位资金130亿元以上。

各位代表，幸福都是奋斗出来的，发展都是实干出来的。我们要始终把实干作为政府自身建设的生命线，用实干践行“四个意识”和“两个维护”。

一是在解放思想中看实干精神。旗帜鲜明讲政治，认真开展“不忘初心、牢记使命”主题教育，让习近平新时代中国特色社会主义思想走心入魂。坚持重大事项、重要工作向市委及时报告，坚决落实市委决策部署。深入开展干部素质提升年活动，改进政府系统学习方式，着力破除畏难等靠的心态、自甘现状的状态、大而化之的习惯，树立起向党中央对标对表的自觉，更加主动地置身全国全区大局中谋划开展工作，创造性抓好落实，用发展实绩、经济质量、办事效率检验学习成效。树立正确的政绩观，谋发展、做决策、干事情始终立足于固原是全域贫困市和发展不充分的最大实际，以“功成不必在我”的精神境界和“功成必定有我”的历史担当，为民多留财富、少留包袱。褒能治慵、奖勤罚懒，以实干“论英雄”。

二是在担当作为中看实干责任。把实干作为基本要求，“实”字当头、“干”字为先。要履行巡视整改政治责任，敬终如始推动巡视督查反馈问题整改见效、固化成果。要履行引领发展主体责任，强化研判经济形势，精准施策，靶向发力。对重点工作、重点项目要细化目标、量化任务、靠实责任、挂图作战，以问责问效、奖优罚劣倒逼工作提质提速提效；在项目谋划上聚力，精心谋划一批产业升级、生态环保、基础设施、防灾减灾等高质量发展重大项目，为投资恢复性增长奠定坚实基础；积极跑部进厅汇报对接，市政府领导每季度至少对接部委1次，县（区）和市直部门每月至少对接厅局1次，全年争取国家、自治区各类资金220亿元；安排项目前期工作经费5000万元，重点保障支撑“六场硬仗”的80个重点建设项目及10个重点前期项目（见附件1）和9项20件民生实事（见附件2）；建立重点项目部门并联审批、明确市领导跟踪协调、督查室专项督办工作机制，集中解决项目前期工作效率低下、要素保障不到位、项目推进缓慢等问题，力争80%的新建项目3月底前开工并高质量推进。

三是在依法履职中看实干本领。坚持运用战略思维、创新思维、辩证思维、法治思维、底线思维，抓重点、难点和关键工作。按照“五个过硬”的要求，在学习实践中练就担当的宽肩膀和成事的真本领。要提高依法行政本领，进一步完善政府议事规则及重大决策程序，健全决策咨询制度，自觉运用法治思维和法治方式解决问题、推动工作；依法接受市人大及其常委会的监督，自觉接受市政协的民主监督，主动接受社会和舆论监督。要提高群众工作本领，建立政策发布、解读、回应、公开机制，畅通群众与政府互动渠道，让人民了解政府、监督政府。要提高狠抓落实本领，坚持到一线指挥，在一线协调，主动服务基层、服务企业、服务群众，推动政府职能从管理向服务深度转变，确保为老百姓承诺的事一件一件落到实处。要提高驾驭风险本领，坚决打好防范化解重大风险攻坚战，严禁违法违规举债和担保行为，确保债务规模逐年下降；严厉打击非法金融活动。

四是在廉洁从政中看实干作风。认真落实十九届中央纪委三次全会、自治区第十二届纪委三次全会精神，深入开展干部作风转变年活动，力戒表态不负责、行动层层拖、雷声大雨点小的形式主义、官僚主义，坚决纠正只挂帅不出征或出兵多战果少的落实，坚决制止只花钱不求效或花钱多绩效差的投入。落实全面从严治党要求，严守政治纪律和政治规矩，严肃党内政治生活；严格执行中央八项规定及实施细则精神和自治区有关规定，从领导干部做起，少开会、开短会、开管用的会，少发文、发短文、发有作用的文，多到基层一线开展蹲点式调研，集中整治“口号式”贯彻落实、“走秀式”调查研究、“照搬式”发文开会等问题。认真落实市政府党组党风廉政建设主体责任和班子成员“一岗双责”，树立过紧日子的思想，今年“三公”经费和一般性支出零增长；强化对涉农、扶贫等重点领域监管，切实解决好损害群众利益问题。加强支持国家监察和审计监督工作，养成在监督下工作的习惯，决不辜负人民的信任和组织的嘱托。

各位代表，人心齐，泰山移。让我们更加紧密地团结在以习近平同志为核心的党中央周围，高举中国特色社会主义伟大旗帜，在自治区党委、政府和市委的坚强领导下，不忘初心、牢记使命，振奋精神，实干兴宁，为“建设美丽新宁夏、共圆伟大中国梦”作出新的更大贡献，以优异成绩向新中国成立70周年献礼!
